# Supplementary material for: Mowing Facilitated Shoot and Root Litter Decomposition Compared with Grazing
Source: Plants (Basel). 2022 Mar 23;11(7):846. doi: 10.3390/plants11070846 (PMC9002786; doi:10.3390/plants11070846)
Supplement: Supplementary file 1 [file plants-11-00846-s001.zip › plants-1643953-supplementary.pdf]

Supplemental information

Table S1. Main species composition of the study plot

Treatment	Main Species	Important Value
Low altitude + Mowing	*Bromus inermis*	0.37±0.03
	Elymus repensc	0.19±0.03
	Medicago sativa	0.18±0.02
	Onobrychis viciifolia	0.07±0.02
Low altitude + Grazing	*Achnatherum inebrians*	0.45±0.05
	Stipa capillata	0.21±0.09
	Chenopodium album	0.14±0.02
	Potentilla chinensis	0.09±0.01
High altitude + Mowing	*Bromus inermis*	0.33±0.03
	Alchemilla tianschanica	0.16±0.05
	Galium linearifolium	0.14±0.04
	Geranium rotundifolium	0.13±0.02
High altitude + Grazing	*Stipa capillata*	0.33±0.02
	Potentilla chinensis	0.20±0.04
	Trifolium repens	0.17±0.03
	Achnatherum inebrians	0.08±0.05

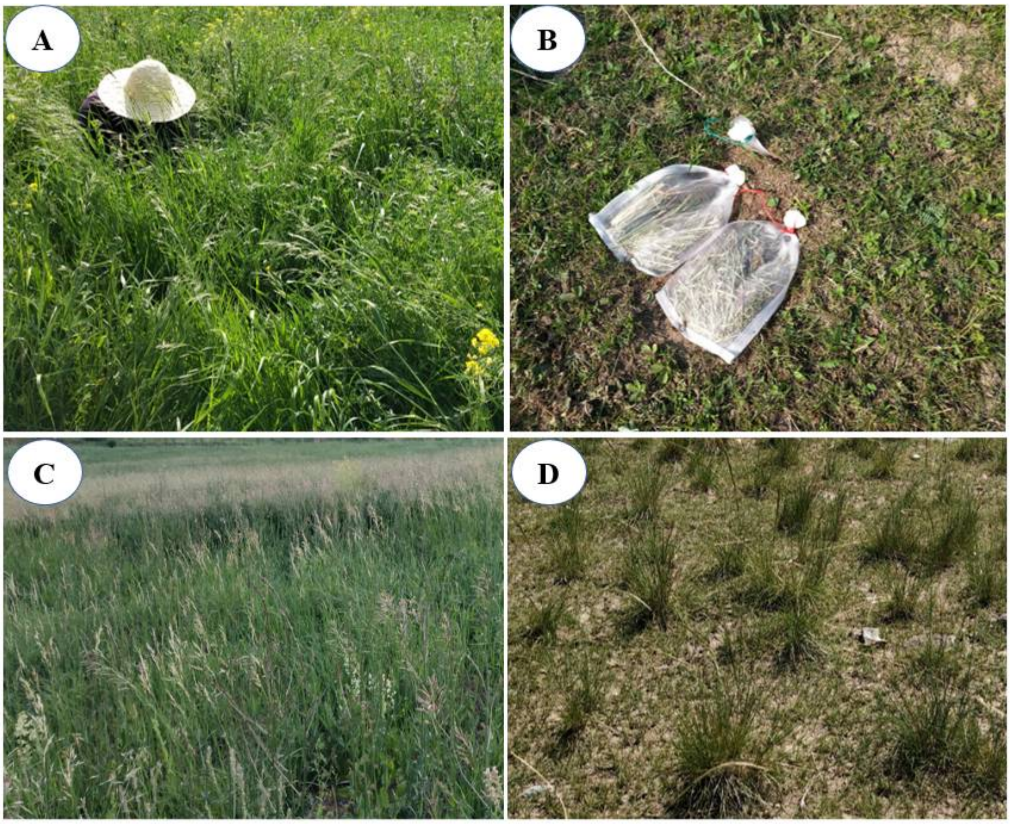

Figure S1. Litter decomposition plot habitat. A, high altitude + mowing; B, high altitude + grazing; C, low altitude +mowing; D, low altitude + grazing.

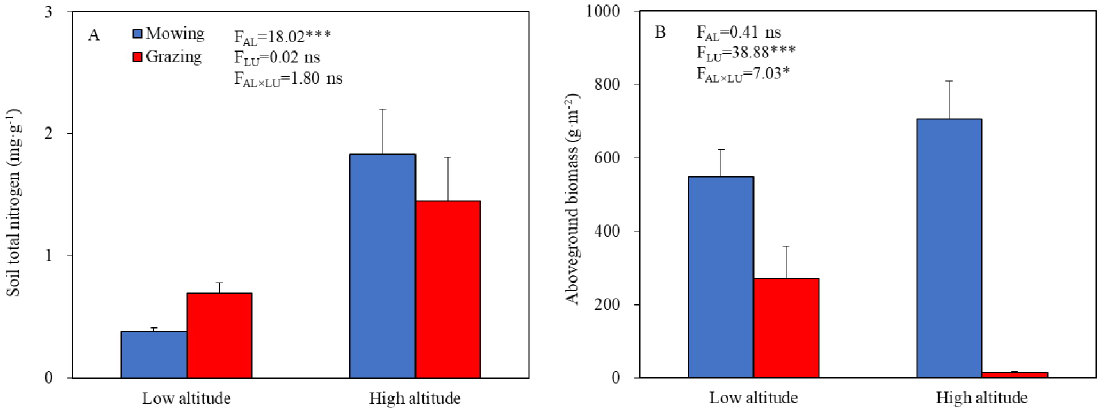

Figure S2. Effects of altitude and land-use type on (A) soil total nitrogen and (B) aboveground biomass (Mean ± SE). The ns, *, and *** represent $p > 0.05$, $p < 0.05$ and $p < 0.001$.

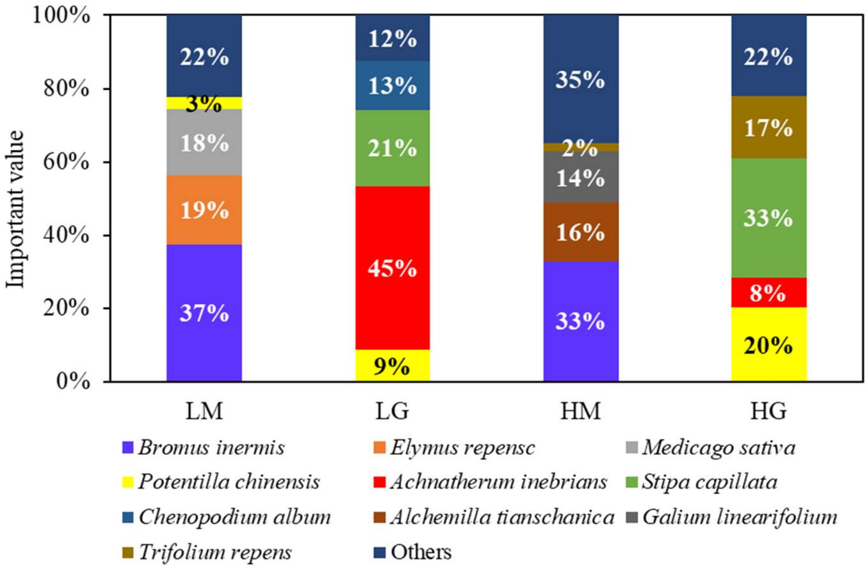

Figure S3. Proportion of important values of various species in different treatments. LM: low altitude + mowing; LG: low altitude + grazing; HM: high altitude + mowing; HG: high altitude + grazing.
